# The association of food quality index with mental health in women: a cross-sectional study

**DOI:** 10.1186/s13104-020-05401-x

**Published:** 2020-12-09

**Authors:** Manije Darooghegi Mofrad, Fereydoun Siassi, Bijan Guilani, Nick Bellissimo, Katherine Suitor, Leila Azadbakht

**Affiliations:** 1 Department of Community Nutrition, School of Nutritional Sciences and Dietetics, Tehran University of Medical Sciences, Tehran, Iran; 2 Department of Clinical Psychology, University of Tehran, Tehran, Iran; 3 School of Nutrition, Ryerson University, Toronto, Canada; 4 Diabetes Research Center, Endocrinology and Metabolism Clinical Sciences Institute, Tehran University of Medical Sciences, Tehran, Iran; 5 Department of Community Nutrition, School of Nutrition and Food Science, Isfahan University of Medical Science, Isfahan, Iran

**Keywords:** Food quality score, Depression, Anxiety, Stress

## Abstract

**Objective:**

Diet is a fundamental modifiable risk factor for the development of depression and anxiety. However, no evidence currently exists regarding the association of food quality score (FQS) and mental health in Iranian women. This study investigated the relationship between food quality score, depression, anxiety and stress in Iranian women.

**Results:**

Results showed that 35%, 41% and 42% of participants had depression, anxiety and stress, respectively. The average age of participants was 31.40 ± 7.47 years. A significant association was indicated between FQS and symptoms of depression (OR: 0.36; 95% CI 0.21, 0.63), anxiety (OR: 0.31; 95% CI 0.18, 0.53), and stress (OR: 0.39; 95% CI 0.23, 0.66) in both crude and adjusted models.

## Introduction

The global burden of depression and anxiety has become prominent in both developed and developing countries [1]. The incidence of depression and anxiety is ~ 5% and ~ 7% worldwide, respectively [2, 3]. In Iran, the prevalence of depression and anxiety among adults is 21% and 20%, respectively, and are twice as likely to happen in Iranian women compared to men [4].

Diet is a substantial modifiable risk factor for the development of depression and anxiety [5]. Researchers have suggested whole diet as an alternative approach to study the potential role of diet for prevention of disease [6, 7]. However, diet quality indices typically require additional analyses to assess nutrient intake, and nutrient databases are commonly based on a Western diet and are not usually validated in other populations. In comparison, food-based scores, such as food quality score (FQS), only considers foods or food groups and does not rely on nutrient analysis [8]. FQS is a relatively new index and has only been used in three studies to examine the association between coronary artery disease [8], metabolic syndrome [9], and cardiovascular risk factors [10]. However, FQS components have been previously related to mental health [11–17], and findings from Mozaffarian et al. demonstrate a significant association between FQS components and long-lasting weight loss [18].

To our knowledge, no evidence currently exists regarding the relationship between FQS and mental health. Therefore, our study was done to assess the association between FQS, depression, anxiety and stress in Iranian women.

## Main text

### Materials and methods

#### Study design and participants

A multistage cluster sampling method was used to recruit 457 women aged 20-50 y from 10 health centers in the south of Tehran, Iran for the current cross-sectional study. Sample size was calculated according to the following formula: N = [(Z_1-α_/2)^2^ P(1-P)]/d^2^](P = 29, d = 4.2 and α = 0.05), P = prevalence of mental disorders [4]. Exclusion criteria included women who had immigrated, were pregnant, postmenopausal, lactating, diagnosed with clinical depression, or were prescribed related medications within the previous 12 months. Further, participants were excluded if they adhered to a specific diet, had experienced an adverse life event in the past 6 months or reported a history of chronic disease (e.g. cancer, diabetes, hypertension, cardiovascular disease, epilepsy, or multiple sclerosis). Verbal consent and written informed consent were collected from each participant.

#### Dietary assessment

A validated 168-item semi-quantitative food frequency questionnaire (FFQ) was used to assess participant dietary intake [19]. An in-person interview was conducted for each participant by a trained nutritionist to record the frequency of consumption for each food item in the previous 12 months. Common household measures were used to estimate portion size (g) and recorded in a modified version of NUTRITIONIST IV software, specific to Iranian food items (version 7.0; N-Squared Computing, Salem, OR, USA) to estimate mean energy and nutrient intake. Participants were excluded if an average energy intake outside 500–3500 kcal/day was reported.

#### FQS calculation

A Food Quality Score (FQS) was derived using the method previously described by Fung et al. [8]. Each individual food item was ranked based on health effects (favourable vs. unfavourable) that had been previously associated with weight change [18]. Healthy food items included nuts and legumes, fruits, vegetables, yogurt, whole grains, and coffee while unhealthy food items included refined grains, sugar sweetened beverages (SSBs), desserts, potatoes, potato chips, red meat, processed meat, fried foods prepared away from home, and. For each food group, the dietary intake of each participant was classified into deciles and assigned a score of 1–10 to food groups previously shown to have favourable health effects, while reverse decile rankings (scores of 10 down to 1) for food groups shown to have unfavourable health effects. The score of each food group was summed to obtain an overall possible score between 14 and 140 (based on a total of 14 food groups) where a higher score represented a healthier diet.

#### Assessment of psychological profile

Depression, anxiety and stress were determined using the Depression Anxiety Stress Scales (DASS-21), previously validated in an Iranian population [20]. The DASS-21 is a self-reported questionnaire that includes 21 items, 7 items for each component: depression, anxiety and stress. Participants were asked to record a score between 0 (did not apply to me at all)-3 (applied to me very much). Scores were summed for each component and multiplied by a factor of 2 as DASS-21 is derived from the DASS-42. Based on cut-off scores by Lovibond et al. [21], participants were categorized into five grades (normal, mild, moderate, severe, and very severe) for stress depression, and anxiety. In the current study, participants were ranked into two groups: normal and persons with depression, anxiety, and stress because of insufficient number of cases in some categories. Participants with score higher than 7, 9, and 14 considered anxious, depressed, and stressed, respectively.

#### Assessment of other variables

An SES score was derived based on the index described in [22]. A total score was determined based on factors including education, family size, job status and occupation, home owning and home equipment diversity, room counts, and domestic/international travel [22]. SES scores were categorized into tertiles where the first tertile was considered low SES and the third tertile was considered high SES. Each participant’s weight (kg) and height (m) were measured and body mass index was estimated by formula (kg/m^2^). The physical activity of each participant was asked over a 24 h period and physical activity level was determined based on multiplying metabolic equivalents (MET) by hours per day (Met * h/d) [23].

#### Statistical analysis

An analysis of variance (ANOVA) was used to compare continuous demographic variables (reported as mean ± standard deviation (SD)) while a Chi square analysis was used to evaluate categorical variables (reported as numbers and percentages) across FQS tertiles. An analysis of covariance (ANCOVA) was applied to assess differences between participant dietary intake across FQS tertiles. All dietary intake data, excluding energy intake, was controlled for energy intake. Binary logistic regressions were used to measure odds ratio (ORs) and 95% CI in crude and multivariable-adjusted models to evaluate the association of FQS with depression, anxiety and stress. In model 1, age and total energy intake were adjusted for. In model 2, age, energy intake, marital status, physical activity, SES, supplement use, medication use, body size satisfaction, family history of chronic disease, time spent outside of the home, duration of sleep were adjusted for. In model 3, BMI was adjusted for in addition to the factors listed in model 2. Statistical analyses were conducted using SPSS (version 21; SPSS Inc., Chicago, IL, USA), with significance defined as *P* < 0.05.

### Results

Based on the DASS-21, the prevalence of depression was 35%, anxiety 41% and stress 42% in our study population. Participant characteristics are provided in Table 1. Subjects in the third tertile of FQS were older, and less likely to be depressed, anxious and stressed. No significant differences were observed in other demographic characteristics across tertiles of FQS.

**Table 1:** General characteristics of participants across the tertiles of FQS

Variable	FQS tertiles				P value^a^
	Total (N = 457)	T1 ≤72 (n = 167)	T2 72 < to 84 (n = 160)	T3 ≥84 (n = 130)	
Age(year)	31.40 ± 7.47^a^	29.53 ± 6.99	31.98 ± 7.63	33.09 ± 7.39	<0.001
BMI (kg/m^2^)	23.92 ± 3.94	23.6 ± 4.31	23.66 ± 3.83	24.61 ± 3.50	0.060
Weight(kg)	62.96 ± 10.50	62.58 ± 11.53	62.34 ± 10.30	64.21 ± 9.26	0.270
Physical activity (Met.h/d)	39.85 ± 6.60	39.06 ± 7.35	40.06 ± 5.48	40.61 ± 6.76	0.117
Sleeping time(hrs)	7.81 ± 1.53	7.94 ± 1.52	7.73 ± 1.53	7.73 ± 1.52	0.376
Out time(hrs)	6.25 ± 3.73	6.57 ± 3.67	6.05 ± 3.76	6.10 ± 3.79	0.393
Socioeconomic status (n (%))					
Low	142 (31)	50 (35)	53 (37)	39 (28)	0.897
Medium	178 (39)	69 (39)	63 (35)	46 (26)	
High	137 (30)	48 (35)	44 (32)	45 (33)	
Overweight or obesity (n (%))	157 (34)	51(32)	54 (34)	52 (34)	0.230
Marital status (n (%))					
Single	187 (40)	73 (39)	65 (35)	49 (26)	0.576
Married	270 (60)	94 (35)	95 (35)	81 (30)	
Education status (n (%))					
≤Diploma	148 (33)	48 (32)	59 (40)	41 (28)	0.283
>Diploma	309 (67)	119 (38)	101 (33)	89 (29)	
Head of household education (n (%))					
≤Diploma	234 (51)	82 (35)	84 (36)	68 (29)	0.792
>Diploma	223 (49)	85 (38)	76 (34)	62 (28)	
Supplement use (n (%))					
Yes	169 (37)	61 (36)	62 (37)	46 (27)	0.830
No	288 (63)	106 (37)	98 (34)	84 (29)	
Drug use (n (%))					
Yes	36 (8)	16 (44)	9 (25)	11 (31)	0.397
No	421 (92)	151 (36)	151 (36)	119 (28)	
Family history of chronic disease (n (%))					
Yes	245 (54)	97 (40)	84 (34)	64 (26)	0.297
No	212 (46)	70 (33)	76 (36)	66 (31)	
Body size satisfaction (n (%))					
Yes	317 (69)	109 (34)	114 (36)	94 (30)	0.347
No	140 (31)	58 (41)	46 (33)	36 (26)	
Depression (n (%))	159 (34.8)	73 (45.9)	54 (34)	32 (20.1)	0.003
Anxiety (n (%))	185 (40.5)	90 (48.6)	58 (31.4)	37 (20.0)	<0.001
Stress (n (%))	192 (42)	92 (47.9)	58 (30.2)	42 (21.9)	<0.001

Values are reported mean ± SD, unless indicated

*FQS* Food Quality Score, *BMI* Body Mass Index, *WC* Waist Circumference

^a^Using one-way ANOVA for continuous variables and Chi square test for categorical variables

Selected food groups and nutrient intake of participants are reported in Table 2. Participants in the third FQS tertile reported significantly greater intake of protein, fiber, folic acid, vitamin A, vitamin B6, vitamin C, magnesium, calcium, potassium, iron and zinc compared to participants in the first tertile. Saturated fat intake was significantly lower in the third tertile of FQS than the first tertile.

**Table 2:** Dietary intakes of study participants across the tertiles of FQS

Variable	FQS tertile			*P* value^a^
	T1 ≤72 (n = 167)	T2 72 < to 84 (n = 160)	T3 ≥84 (n = 125)	
Energy (kcal/d)	2130.83 ± 38.69	2052.39 ± 39.53	2030.82 ± 43.85	0.182
Protein (g/day)	72.93 ± 1.12	74.59 ± 1.14	79.49 ± 1.27	<0.001
Carbohydrate (g/day)	284.20 ± 2.43	292.34 ± 2.48	289.38 ± 2.75	0.062
Fat (g/day)	77.45 ± 1.05	74.53 ± 1.07	75.72 ± 1.18	0.150
Dietary fiber (g/d)	13.77 ± 0.33	16.76 ± 0.34	24.41 ± 0.37	<0.001
SFA (g/day)	24.30 ± 0.44	22.78 ± 0.45	22.28 ± 0.50	0.006
PUFA (g/day)	16.93 ± 0.39	16.14 ± 0.40	16.47 ± 0.44	0.364
MUFA (g/day)	22.58 ± 0.46	22.06 ± 0.47	22.59 ± 0.52	0.671
Cholesterol (mg/day)	224.00 ± 6.21	216.58 ± 6.33	221.62 ± 7.03	0.697
Folic acid (μg/day)	276.17 ± 5.88	316.94 ± 5.99	372.25 ± 6.65	<0.001
Vitamin A (RAE/day)	1111.42 ± 58.65	1323.85 ± 59.80	1707.16 ± 66.39	<0.001
Thiamine (mg/day)	1.51 ± 0.01	1.52 ± 0.01	1.55 ± 0.02	0.414
Vitamin B6 (mg/day)	1.25 ± 0.026	1.31 ± 0.027	1.45 ± 0.030	<0.001
Vitamin B12 (μg/day)	4.58 ± 0.19	4.59 ± 0.19	4.48 ± 0.22	0.923
Vitamin C (mg/day)	114.74 ± 4.69	141.64 ± 4.78	168.83 ± 5.31	<0.001
Calcium (mg/day)	969.16 ± 21.27	1031.07 ± 21.68	1158.50 ± 24.08	<0.001
Magnesium (mg/day)	241.77 ± 3.53	265.12 ± 3.60	299.07 ± 4.00	<0.001
Potassium (mg/day)	2993.06 ± 54.31	3414.10 ± 55.37	3876.17 ± 61.48	<0.001
Zinc (mg/day)	8.50 ± 0.22	8.79 ± 0.22	9.85 ± 0.25	<0.001
Fe (mg/d)	19.14 ± 1.60	21.71 ± 1.69	24.98 ± 1.68	0.043
Fruit (g/day)	236.61 ± 14.29	319.20 ± 14.57	368.22 ± 16.17	<0.001
Vegetable (g/day)	265.54 ± 13.70	352.59 ± 13.97	461.94 ± 15.51	<0.001
Nuts and legumes (g/day)	40.32 ± 2.77	54.84 ± 2.83	72.65 ± 3.14	<0.001
Whole grains (g/day)	7.56 ± 0.95	8.90 ± 0.97	15.27 ± 1.07	<0.001
Yogurt (g/day)	143.79 ± 8.08	178.68 ± 8.24	183.52 ± 9.15	0.001
coffee (g/day)	25.67 ± 6.05	35.47 ± 6.17	60.62 ± 6.85	0.001
Refined grains (g/day)	332.93 ± 7.75	309.20 ± 7.90	263.68 ± 8.77	<0.001
Red meat (g/day)	46.56 ± 2.62	40.30 ± 2.67	40.10 ± 2.97	0.158
Processed meat (g/day)	11.13 ± 0.64	5.74 ± 0.66	3.62 ± 0.73	<0.001
Desserts and ice cream (g/day)	105.31 ± 3.40	88.63 ± 3.46	68.65 ± 3.84	<0.001
Potato (g/day)	25.78 ± 1.63	24.43 ± 1.66	20.82 ± 1.84	0.124
Potato chips (g/day)	6.14 ± 0.50	4.16 ± 0.51	1.94 ± 0.56	<0.001
Sugar sweetened beverage (g/day)	30.10 ± 3.75	25.58 ± 3.83	15.06 ± 4.25	0.028
Fried food from outside the home (g/day)	21.91 ± 1.44	13.97 ± 1.46	11.41 ± 1.63	<0.001

Values are mean ± SE. All values are adjusted for energy intake, except for total energy intake

*FQS* Food Quality Score, *SFA* saturated fatty acid, *PUFA* poly unsaturated fatty acid, *MUFA* mono unsaturated fatty acid, *RAE* Retinol activity equivalents

^a^Using ANCOVA

Multivariable-adjusted ORs for depressive symptoms, anxiety and stress across FQS tertiles are reported in Table 3. After adjustment for potential confounders, our findings suggest women in the third tertile of FQS had a significantly lower risk for depression, anxiety and stress compared to participants in the first tertile.

**Table 3:** Multiple-adjusted odds ratio (OR) and 95% confidence intervals (CI) for psychological disorders based on categories of FQS

Variable	FQS tertiles			*P* trend^e^
	T1 ≤72 (n = 167)	T2 72 < to 84 (n = 160)	T3 ≥ 84 (n = 130)	
Depression				
Crude	1.00	0.65 (0.41, 1.02)	0.42 (0.25, 0.69)^d^	0.001
ModeL I^A^	1.00	0.55 (0.34, 0.88)	0.32 (0.19, 0.55)	<0.001
Model II^B^	1.00	0.59 (0.36, 0.96)	0.36 (0.21, 0.62)	<0.001
Model III^C^	1.00	0.59 (0.36, 0.96)	0.36 (0.21, 0.63)	<0.001
Anxiety				
Crude	1.00	0.48 (0.31, 0.75)	0.34 (0.20, 0.55)	<0.001
Model I	1.00	0.44 (0.27, 0.69)	0.29 (0.17, 0.44)	<0.001
Model II	1.00	0.46 (0.29, 0.74)	0.31 (0.18, 0.53)	<0.001
Model III	1.00	0.46 (0.29, 0.74)	0.31 (0.18, 0.53)	<0.001
Stress				
Crude	1.00	0.46 (0.29, 0.72)	0.38 (0.24, 0.62)	<0.001
Model I	1.00	0.42 (0.27, 0.67)	0.34 (0.21, 0.56)	<0.001
Model II	1.00	0.45 (0.28, 0.73)	0.38 (0.23, 0.65)	<0.001
Model III	1.00	0.48 (0.30, 0.77)	0.39 (0.23, 0.66)	<0.001

*FQS* Food Quality Score

^a^Adjusted for age and energy intake

^b^Additionally adjusted for SES, physical activity, marriage status, supplement use, drug use, family history of chronic disease, satisfaction with the physical form, number of sleeping hours, and number of outside hours

^c^Additionally adjusted for BMI

^d^These values are odds ratios (95% CIs)

^e^Obtained from logistic regression by considering tertiles of FQS as ordinal variable

### Discussion

Findings from our cross-sectional study provide evidence that before and after controlling for potential confounding variables, higher FQS is associated with significantly lower risk for depression, anxiety and stress among Iranian women. To our knowledge, this is the first study assessing the association between FQS and mental health in Iranian women.

Dietary patterns have been previously linked to depression, anxiety and stress but the evidence remains equivocal. A healthy dietary pattern characterized by vegetables, fruit, whole grains, monounsaturated fat and fish has been associated with a reduced risk for depression [24], anxiety [25] and stress [26]. In comparison, the prevalence of depression and anxiety has been previously linked to a Western dietary pattern rich in processed foods, SSBs, animal protein and refined grains [27]. Yet, a recent systematic review provided evidence which indicated no significant association between the Western diet and depression [28]. While other studies have reported dietary scores including the Dietary Approaches to Stop Hypertension (DASH)-score, dietary inflammatory index and dietary diversity score were not associated with stress [29–31]. This is likely a reflection of the different methods and populations used to assess dietary intake and psychological symptoms.

The relationship between diet and mental health is extremely complex, therefore probable mechanisms for the relationship between FQS and depression, anxiety and stress are unclear. However, this relationship is likely the result of the synergistic effects of all dietary components evaluated in the FQS instead of each nutrient or food group. For example, the high content of antioxidants in fruits and vegetables may confer beneficial protective effects against depression through reduced levels of oxidative stress and neuronal damage [32, 33]. Nut consumption may decrease depressive symptoms by regulating neurotransmitters [34], reducing inflammation [35] or alterations in the central nervous system [36]. Additionally, the high caffeine content in coffee may stimulate the central nervous system and improve dopaminergic neurotransmission [37]. Further, recent evidence has suggested a link between gut microbiome health and the brain. Indeed, yogurt consumption may increase beneficial gut bacteria [38] and reduce symptoms of depression [39]. In contrast, a Western dietary pattern characterized by the consumption of foods with a high glycemic index may lead to poor blood glucose regulation, increase oxidative stress and influence the development of psychological symptoms [40]. Further, a dietary pattern high in sugar and fat (e.g. processed meats and refined grains) has been associated with low-grade inflammation throughout the body characterized by increased levels of C-reactive protein (CRP). Not only is CRP an acute-phase inflammatory marker but can indicate an increased risk for depression [40].

### Conclusion

Our findings suggest a higher food quality score was significantly associated with a lower risk of depression, anxiety and stress. Future prospective studies are required to affirm our findings.

### Limitations

The cross-sectional design limited our ability to determine the cause-effect relationship between FQS and mental health. Additionally, our study used the DASS-21, a self-reported questionnaire, rather than a psychiatrist to assess symptoms of depression, anxiety and stress. Further, the effect of residual socioeconomic, behavioural or lifestyle confounding variables may explain some of our findings. Finally, our study findings cannot be generalized; the current study was conducted using Iranian women aged 20–50 y who attended the health centers in our catchment area. To our knowledge, this relationship has not been explored in men within the same age category and women in other age categories.

## Data Availability

Data available upon reasonable request due to privacy/ethical considerations.

## Abbreviation

BMI: Body mass index
CI: Confidence interval
DASH: Dietary Approaches to Stop Hypertension
DASS: Depression, Anxiety Stress Scales
FQS: Food quality score
FFQ: Food frequency questionnaire
OR: Odds ratio
SSBs: Sugar-sweetened beverages
SES: Socioeconomic status
